# Complete Genome Sequences of Sequence Type 71 (ST71) and ST97 Staphylococcus aureus Isolates from Bovine Milk

**DOI:** 10.1128/MRA.00954-18

**Published:** 2018-08-09

**Authors:** Paul Cormican, Orla M. Keane

**Affiliations:** a Animal & Bioscience Department, Teagasc, Grange, Dunsany, Co. Meath, Ireland; University of Arizona

## Abstract

This is the announcement of draft genome sequences for Staphylococcus aureus strains belonging to sequence type 97 (ST97) and ST71. These sequence types are commonly associated with bovine mastitis, and the strains were isolated in Ireland in 2010 from the milk of cows with clinical mastitis.

## ANNOUNCEMENT

Staphylococcus aureus is a common cause of bovine mastitis and is frequently associated with subclinical infections that are refractory to treatment (1). A number of bovine-adapted S. aureus lineages are responsible for the majority of cases of bovine mastitis worldwide (2). Livestock-associated S. aureus is also a public health concern due to the potential for zoonotic transfer and the ability to act as a reservoir of antimicrobial resistance determinants (3). Sequence type 97 (ST97) is the founder of clonal complex 97 (CC97), a globally distributed bovine-adapted lineage which has increasingly been associated with human infections (4). A subgroup of CC97, founded by ST71, was recently described. Isolates in this subgroup have undergone a genomic rearrangement of over 300 kb in the region of the origin of replication (5) and differ in their expression of some virulence traits (6). Complete genome sequences of ST71 and ST97 will contribute to comparative genomics studies of bovine-adapted S. aureus and to clearly defining the breakpoints between ST71 and ST97.

S. aureus strains MOK042 (ST71) and MOK063 (ST97), each isolated from a single colony, were grown overnight in 4 ml of Trypticase soy broth at 37°C. The bacterial pellet was subsequently resuspended in 3.5 ml of Qiagen buffer B2 containing 87.5 µl RNase A (4 mg/ml; Promega); 80 µl lysostaphin (10 mg/ml; Sigma-Aldrich) and 100 µl proteinase K stock solution (Qiagen) were added, followed by incubation at 37°C for 1 h. Genomic DNA was then extracted using the Qiagen Genomic-tip 100/G kit according to the manufacturer’s protocol.

Whole-genome sequences were generated by the Earlham Institute (Norwich, UK) using one single-molecule real-time (SMRT) cell of a PacBio RS II sequencer (Pacific Biosciences). *De novo* assembly utilizing Hierarchical Genome Assembly Process 3 (HGAP3) (Pacific Biosciences) yielded two circular contigs for MOK042 and one circular contig for MOK063, with 216- and 259-fold coverage, respectively. The mean subread lengths for MOK042 and MOK063 were 8,682 and 9,209, respectively, and the G+C content of each isolate was 32.8%. The assembled genome sequences of S. aureus MOK042 and MOK063 are 2,844,513 bp and 2,808,798 bp long, respectively, while the second contig of MOK042 encoded a plasmid of 31,625 bp displaying 98% sequence identity (over 90% coverage) with plasmid pWBG707 from S. aureus WBG7410 (7). The Prokaryotic Genome Annotation Pipeline (PGAP) at NCBI was used to predict 3,006 genes and 2,813 protein coding sequences for S. aureus MOK042 and 2,907 genes and 2,722 protein coding sequences for S. aureus MOK063 (8). The antibiotic resistance and virulence gene profiles of these isolates have been previously described (5). Three incomplete and two intact (49.3 and 46.5 kb) prophages were identified in the genome of MOK042, and five incomplete and one intact (49.3 kb) prophages were identified in the genome of MOK063 using the PHAge Search Tool (PHAST) (9). Strain MOK063 also displayed a 1.72-Mbp genomic inversion relative to MOK042 and RF122 (10).

### Data availability.

This complete genome project has been deposited in GenBank under the accession numbers CP029627, CP029628, and CP029629.

## References

[1] Keane OM, Budd KE, Flynn J, McCoy F 2013 Pathogen profile of clinical mastitis in Irish milk-recording herds reveals a complex aetiology. Vet Rec 173:17. doi:10.1136/vr.101308. 23694921

[2] Smyth DS, Feil EJ, Meaney WJ, Hartigan PJ, Tollersrud T, Fitzgerald JR, Enright MC, Smyth CJ 2009 Molecular genetic typing reveals further insights into the diversity of animal-associated *Staphylococcus aureus*. J Med Microbiol 58:1343–1353. doi:10.1099/jmm.0.009837-0. 19528163

[3] Fitzgerald JR 2012 Livestock-associated *Staphylococcus aureus*: origin, evolution and public health threat. Trends Microbiol 20:192–198. doi:10.1016/j.tim.2012.01.006. 22386364

[4] Spoor LE, McAdam PR, Weinert LA, Rambaut A, Hasman H, Aarestrup FM, Kearns AM, Larsen AR, Skov RL, Fitzgerald JR 2013 Livestock origin for a human pandemic clone of community-associated methicillin-resistant *Staphylococcus aureus*. mBio 4:e00356-13. doi:10.1128/mBio.00356-13. 23943757 PMC3747577

[5] Budd KE, McCoy F, Monecke S, Cormican P, Mitchell J, Keane OM 2015 Extensive genomic diversity among bovine-adapted *Staphylococcus aureus*: evidence for a genomic rearrangement within CC97. PLoS One 10:e0134592. doi:10.1371/journal.pone.0134592. 26317849 PMC4552844

[6] Budd KE, Mitchell J, Keane OM 2016 Lineage associated expression of virulence traits in bovine-adapted *Staphylococcus aureus*. Vet Microbiol 189:24–31. doi:10.1016/j.vetmic.2016.04.013. 27259823

[7] Udo EE, Wei MQ, Grubb WB 1992 Conjugative trimethoprim resistance in *Staphylococcus aureus*. FEMS Microbiol Lett 76:243–248. 1427013 10.1016/0378-1097(92)90343-m

[8] Tatusova T, DiCuccio M, Badretdin A, Chetvernin V, Nawrocki EP, Zaslavsky L, Lomsadze A, Pruitt KD, Borodovsky M, Ostell J 2016 NCBI Prokaryotic Genome Annotation Pipeline. Nucleic Acids Res 44:6614–6624. doi:10.1093/nar/gkw569. 27342282 PMC5001611

[9] Zhou Y, Liang Y, Lynch KH, Dennis JJ, Wishart DS 2011 PHAST: a fast phage search tool. Nucleic Acids Res 39:W347–W352. doi:10.1093/nar/gkr485. 21672955 PMC3125810

[10] Herron-Olson L, Fitzgerald JR, Musser JM, Kapur V 2007 Molecular correlates of host specialization in *Staphylococcus aureus*. PLoS One 2:e1120. doi:10.1371/journal.pone.0001120. 17971880 PMC2040198

